# *molBV* reveals immune landscape of bacterial vaginosis and predicts human papillomavirus infection natural history

**DOI:** 10.1038/s41467-021-27628-3

**Published:** 2022-01-11

**Authors:** Mykhaylo Usyk, Nicolas F. Schlecht, Sarah Pickering, LaShanda Williams, Christopher C. Sollecito, Ana Gradissimo, Carolina Porras, Mahboobeh Safaeian, Ligia Pinto, Rolando Herrero, Howard D. Strickler, Shankar Viswanathan, Anne Nucci-Sack, Angela Diaz, Costa Rica HPV Vaccine Trial (CVT) Group, Bernal Cortés, Paula González, Silvia E. Jiménez, Ana Cecilia Rodríguez, Allan Hildesheim, Aimée R. Kreimer, Douglas R. Lowy, Mark Schiffman, John T. Schiller, Mark Sherman, Sholom Wacholder, Troy J. Kemp, Mary K. Sidawy, Wim Quint, Leen-Jan van Doorn, Linda Struijk, Joel M. Palefsky, Teresa M. Darragh, Mark H. Stoler, Robert D. Burk

**Affiliations:** 1 Department of Pediatrics (Genetic Medicine), Albert Einstein College of Medicine, Bronx, USA; 2 Department of Epidemiology and Population Health, NYU School of Medicine, New York, USA; 3 Department of Epidemiology and Population Health, Albert Einstein College of Medicine, Bronx, USA; 4 Department of Cancer Prevention & Control, Roswell Park Comprehensive Cancer Center, Buffalo, NY USA; 5 Department of Pediatrics, Mount Sinai Adolescent Health Center, Icahn School of Medicine at Mount Sinai, Manhattan, NY USA; 6 Agencia Costarricense de Investigaciones Biomédicas, Fundación INCIENSA, Costa Rica, USA; 7 Roche Molecular Diagnostics, Pleasanton, CA USA; 8 HPV Serology Laboratory, Frederick National Laboratory for Cancer Research, Fredrick, MD USA; 9 Departments of Microbiology and Immunology, and Obstetrics and Gynecology and Women’s Health, Albert Einstein College of Medicine, Bronx, NY USA; 10 United States National Cancer Institute, Bethesda, MD USA; 11 Leidos Biomedical Research, Inc., Frederick National Laboratory for Cancer Research (HPV Immunology Laboratory), Frederick, MD USA; 12 Georgetown University, Washington, DC USA; 13 DDL Diagnostic Laboratory (HPV DNA Testing), Rijswijk, Netherlands; 14 University of Virginia, Charlottesville, VA USA

**Keywords:** Prognostic markers, Molecular medicine, Cancer models, Cancer epidemiology, Metagenomics

## Abstract

Bacterial vaginosis (BV) is a highly prevalent condition that is associated with adverse health outcomes. It has been proposed that BV’s role as a pathogenic condition is mediated via bacteria-induced inflammation. However, the complex interplay between vaginal microbes and host immune factors has yet to be clearly elucidated. Here, we develop *molBV*, a 16 S rRNA gene amplicon-based classification pipeline that generates a molecular score and diagnoses BV with the same accuracy as the current gold standard method (i.e., Nugent score). Using 3 confirmatory cohorts we show that *molBV* is independent of the 16 S rRNA region and generalizable across populations. We use the score in a cohort without clinical BV states, but with measures of HPV infection history and immune markers, to reveal that BV-associated increases in the IL-1β/IP-10 cytokine ratio directly predicts clearance of incident high-risk HPV infection (HR = 1.86, 95% CI: 1.19-2.9). Furthermore, we identify an alternate inflammatory BV signature characterized by elevated TNF-α/MIP-1β ratio that is prospectively associated with progression of incident infections to CIN2 + (OR = 2.81, 95% CI: 1.62-5.42). Thus, BV is a heterogeneous condition that activates different arms of the immune response, which in turn are independent risk factors for HR-HPV clearance and progression. Clinical Trial registration number: The CVT trial has been registered under: NCT00128661.

## Abstract (web summary)

Here, Burk et al. develop an algorithm to diagnose bacterial vaginosis (BV) using the 16S rRNA gene, called *molBV*, which they use to profile the inflammatory landscape of BV and predict progression of human papillomavirus infection to cervical pre-cancer.

## Introduction

Bacterial vaginosis (BV) is defined as vaginal dysbiosis with inflammation and accompanying symptoms including vaginal discharge^1–3^. According to Centers for Disease Control and Prevention (CDC) and NHANES studies, the prevalence of BV is 29.2% amongst reproductive-aged women living in the United States^4,5^. Globally this condition is estimated to have an economic burden of approximately $5-billion per year^6,7^.

In addition to its ubiquity, BV is a urogenital condition that has been associated with adverse reproductive health outcomes including infertility^8^, increased risk for pre-term birth^9^, and low birth weights^10^. Moreover, an active state of BV is associated with an elevated risk for transmission of a variety of sexually transmitted infections (STIs) ranging from bacterial pathogens such as *Chlamydia*^11^ and *Mycoplasma*^12^, to viral agents including HIV^13,14^ and human papillomavirus (HPV)^15^. There is increasing interest in understanding the relationship between the cervicovaginal microbiome (CVM) and HPV natural history and progression to cancer^16–20^. In fact, differences in the CVM might explain why some high-risk HPV (HR-HPV) infections resolve, while others persist and progress. Lastly, BV is also associated with non-reproductive health issues such as obesity^21^.

Clinical BV is primarily diagnosed using Amsel criteria^22^, which requires the presence of three out of four signs or symptoms: (1) homogeneous, thin, white discharge that smoothly coats the vaginal walls; (2) clue cells in a wet mount; (3) pH of vaginal fluid >4.5; and (4) a fishy odor from the vaginal discharge before or after addition of 10% KOH (i.e., whiff test). Although commonly used, this approach has been widely criticized for a considerable rate of misdiagnosis^23^. An alternative to Amsel’s criteria is the Nugent score that creates a composite score based on counts of key bacteria morphologically identified on a Gram stain (i.e., *Lactobacillus*, *Gardnerella,* and curved Gram-negative rods)^24^. Although this method is more sensitive than the Amsel criteria^24–26^, it has been shown to suffer from interobserver variability^27^ and its use has primarily been limited to research settings due to the amount of time, expertise, and costs required to perform the test^28^. The term, molecular BV has been introduced recently^14^ and there are various meanings depending on the system used for molecular detection and the correlation with clinical, bacteriologic, and/or microscopic BV^2,28^. It specifically refers to suboptimal states of the CVM that are usually associated with reduced levels of *Lactobacillus* as measured by molecular techniques.

Bacterial vaginosis has features of an inflammatory state and is associated with alterations of cervicovaginal cytokines^29–32^. A number of studies have reported the association of elevated IL-1β and BV^31,32^, whereas most immune markers associated with BV appear to differ across studies. It has been proposed that this variability may be due to small sample sizes, heterogeneity of study populations^33^, and/or differing microbial taxa within the CVM. It is important to identify the source of this variability since the pathogenic effects of BV appear to be associated with local inflammation^14,32,34–36^.

In this study, we describe a 16S rRNA gene amplicon sequencing-based algorithm, called *molBV*, that can reproducibly categorize BV using a Nugent-like 0-10 score across a variety of populations including those from the US and Africa. Using this molecular approach to identify BV, we report the association of a set of cervicovaginal cytokines with *molBV-*BV. In particular, we demonstrate that elevated levels of *Lactobacillus iners* may in part explain the detection of a BV-like inflammatory signature amongst *molBV*-BV negative women. Although there appears to be a predominant host immune response to *molBV-*BV, the CVM’s positive associations with alternative forms of inflammation are associated with specific microbial agents. We utilize these observations to explore risk factors for the rate of clearance and progression of oncogenic HPV. We provide evidence that an inflammatory cervical profile underlies the association of HR-HPV natural history with *molBV-*BV. Surprisingly, the alternative inflammatory pathway is associated with the progression of HR-HPV infections to neoplastic lesions. This study provides evidence of multiple host inflammatory pathways associated with the cervicovaginal microbiome that influence the outcome of cervicovaginal HPV infection and possibly other pathologic outcomes of bacterial vaginosis.

## Results

### Developing a molecular bacterial vaginosis scoring system

Initially, 30 young women with and without symptoms of BV were recruited for evaluation of Nugent, Amsel and 16S amplicon sequencing. Three samples were inadequate for study leaving a training set of 57 participants (mean age = 21, range 15–25 years). Based on Amsel’s criteria, 22 were classified as BV-positive; whereas, Nugent score evaluations categorized 26 with BV, 8 as intermediate for BV, and 23 as inconsistent with BV (Supplementary Table 1).

**Fig. 1:**
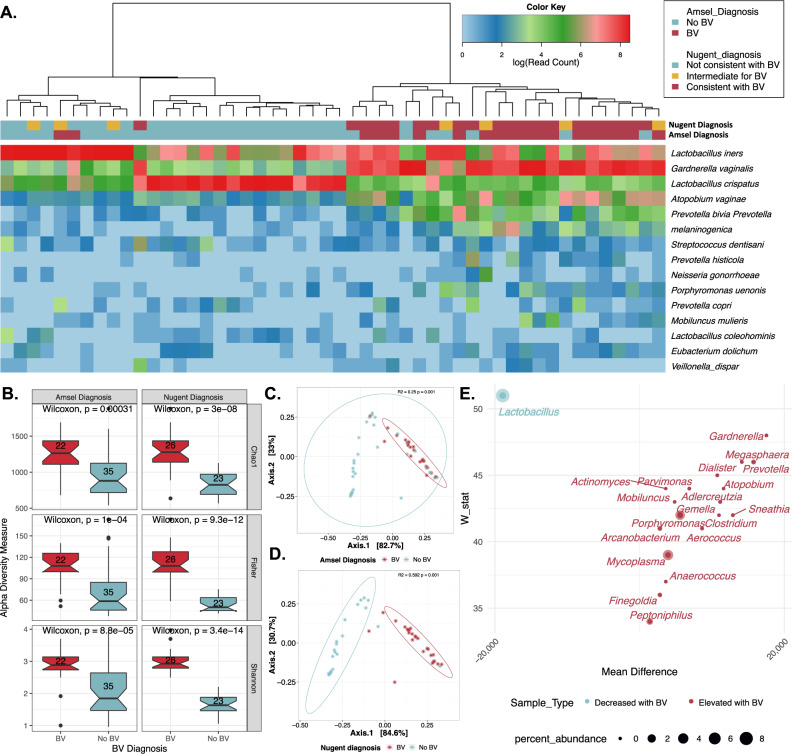
Microbial community features of bacterial vaginosis. Panel **A** shows a heatmap of the 15 most prevalent bacterial species that are indicated to the right of the heatmap. Each column represents a participant. Hierarchical clustering separates samples into two primary clades: one dominated by *Lactobacillus* and one with polymicrobialism. There is a significant tendency of the BV-positive cases to be found in the polymicrobial clade and BV-negative in the one dominated by *Lactobacillus* based on either the Amsel or Nugent diagnosis (see the “Methods” section) (*p* < 0.001 for both). Panel **B** shows the alpha diversity differences between BV diagnosed by either Amsel or Nugent criteria and the microbial communities based on the Chao1, Fisher, and Shannon diversity indices (all *p* < 0.001), as indicated at the right of the panels. Panels **C** and **D** show beta diversity analyses using PCoA and the Jensen–Shannon diversity index for the Amsel BV (panel **C**) (*R*^2^ = 0.25, *p* < 0.001) and Nugent BV diagnosis (panel **D**) (*R*^2^ = 0.59, *p* < 0.001). Panel **E** shows the top 20 microbial markers (based on W-stat) for detecting BV using the “clean” BV status sample set (Amsel+/Nugent+ vs. Amsel−/Nugent−). The *y*-axis represents the ANCOM W-stat, while the *x*-axis represents the mean relative abundance difference between BV+ and BV− cases for each bacterial taxon. The size of the circles represents relative abundance. Source data are provided as a Source Data file.

The 16S rRNA gene V4 region was amplified from cervicovaginal samples of all 57 participants, as it has been shown to robustly detect bacterial species from the cervicovaginal region^37^. There was an average (SD) of 16,580 (487) 16S reads per sample. Fungal sequencing of an ITS1 region amplicon using recently validated primers^38^ resulted in an average (SD) of 16,290 (3486) ITS reads per sample. Following taxonomic assignments and clustering by Euclidean distances (Fig. 1A), samples formed two primary clusters that were either defined by a dominance of two major species from the genus *Lactobacillus* (*n* = 27) or a state of polymicrobialism (*n* = 30). There was a highly significant tendency of the BV-positive samples to sort to the polymicrobial clade and BV negative samples to the *Lactobacillus* clade based on either the Amsel (*p* < 0.001) or Nugent BV diagnosis (*p* < 0.001). Hierarchical clustering using fungal communities revealed two primary clades: one dominated by *Candida albicans*, the other with a dominance of *Malassezia restricta* (Supplementary Fig. 1). The fungal community clustering showed no significant association with binary BV diagnosis, although some clustering was observed for both the Nugent (*p*-value = 0.18) and Amsel BV-positive samples (*p*-value = 0.22).

All alpha diversity measures (Chao1, Fisher, and Shannon, all *p* < 0.001) were highly associated with both Amsel and Nugent outcomes of BV (Fig. 1B). Beta diversity analyses using Jensen–Shannon Divergence (JSD) distances subsampled to 10,000 reads revealed that both the Amsel and Nugent criteria for BV were also significantly associated with the vaginal microbiome *R*^2^ = 0.25, *p* < 0.001 (Fig. 1C) and *R*^2^ = 0.59, *p* < 0.001 (Fig. 1D), respectively. To identify specific taxa associated with BV using ANCOM, we focused on the samples with concordant results for BV by Amsel and Nugent criteria (Supplementary Table 1); 52 differentially abundant genera were identified (FDR < 0.05, Fig. 1E), with *Lactobacillus* being the dominant genus elevated in BV-negative women and a mixture of anaerobic Gram-negative bacteria such as *Gardnerella* elevated in BV-positive, as expected^39–41^. There were no significant associations of fungal alpha or beta measures or specific fungal taxa identified with BV states (Supplementary Fig. 2A–C).

We sought to define a single molecular score from the 16S rRNA gene amplicon next-generation sequencing (NGS) data that would maximize generalizability of such a metric. Thus, we limited the markers to those taxa present in > 80% of all samples at a relative abundance of ≥ 0.001% after subsampling to 10,000 reads. We identified 11 genera meeting these criteria including *Lactobacillus, Prevotella, Gardnerella, Megasphaera, Parvimonas, Clostridium, Porphyromonas, Adlercreutzia, Dialister, Atopobium*, and *Sneathia*. We then derived a *molBV* score using robust regression modeling as described in the “Methods” section and created an averaged score of microbial taxa ratios for each sample providing a score of 0–10 similar to a Nugent score output.

### *molBV* prediction of Nugent BV using three independent clinical datasets

*molBV* was evaluated in publicly available datasets that included 16S rRNA gene amplicon NGS data and measures of bacterial vaginosis. One testing set contained 388 American women with available 16S data sequenced using the V1–V2 16S rRNA gene region (different from the V4 region used above) and clinical Nugent scores^40^. In addition, we identified two African populations, one collected in Cape Town (*n* = 90) and the other in Soweto (*n* = 78) that sequenced the V4 region of 16S and had Nugent measures of BV^42^. We ran the 16S amplicon NGS reads through the *molBV* pipeline to generate Nugent-like scores and observed a strong correlation between the clinical Nugent scores and the *molBV* scores in all three cohorts with ICC values between 0.71–0.81 (Supplementary Fig. 3A). We next assessed the *molBV* score as a discriminant tool for BV diagnostic categories similar to Nugent scores (BV-negative = 0–3 or BV-positive = 7–10)^43^. The *molBV* score showed high AUC values (0.88–0.98) in all three datasets and outperformed other measures of the microbiome such as alpha diversity measures Chao1 and Shannon and the relative abundance of *Lactobacillus* (Supplementary Fig. 3B-D). Thus, the *molBV* pipeline is a robust tool to convert 16S NGS data into BV categories independent of 16S amplicon region and population characteristics.

### The inflammatory landscape of BV

**Fig. 2:**
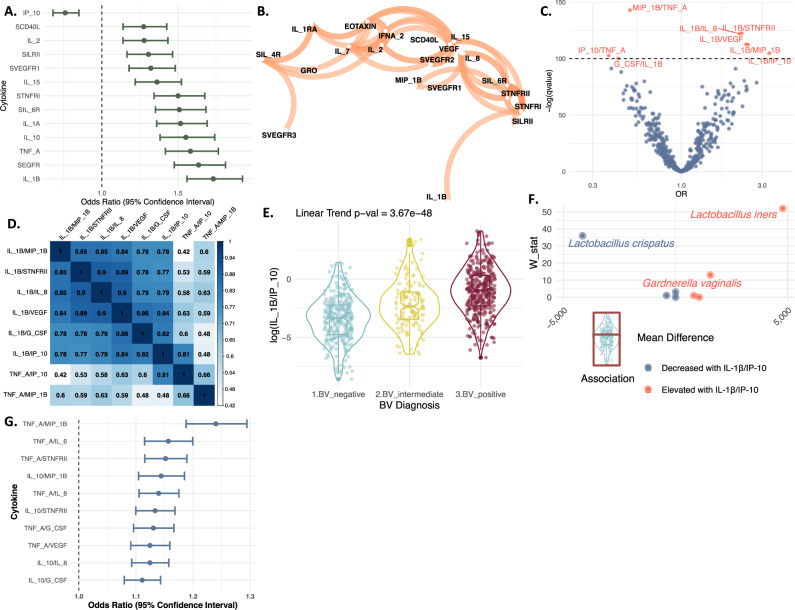
Cytokines associated with *molBV* categorical states in the CVT dataset. Panel **A** forest plot showing the OR and 95% confidence intervals computed using a linear model between the cytokine quartiles and the three ordinal states of *molBV* (i.e., BV-negative, BV-intermediate, and BV-positive) derived using 431 biologically independent CVM samples. Only cytokines with an adjusted *q*-value < 0.001 are presented. ORs in panel **A** represent the odds of moving to the immediate next ordinal BV state. Panel **B** shows the correlation network between all cytokines with a Pearson correlation >0.6 using all baseline samples. Panel **C** is a volcano plot showing the ORs on the *x*-axis when comparing molecular BV-negative vs. BV-positive and the –log(*q*-value) on the *y*-axis. The ratios that had a *q*-value < 3.77*10^-44^ (i.e., −log(*q*-value)>100) are indicated and labeled in red. Panel **D** shows the pairwise Pearson correlation of the highly significant ratios presented in panel **C** (colored red). Ratios that had an OR < 1.0 were inverted for symmetry of data presentation. Clusters from the strongest *molBV*-associated cytokine ratios appear to fall into two primary groups; ones that include IL-1β and those with TNF-α. Panel **E** presents a box and violin plot for the log(IL-1β/IP-10) ratio for BV-negative (colored in blue), BV-intermediate (colored in yellow), and BV-positive (colored in red) samples. Panel **F** shows the bacterial species identified by ANCOM predicting IL-1β/IP-10 inflammation (i.e., above the median) in women within the BV-negative group shown in the small figure below with a red border. Comparison is between samples above the median vs. below the median. Panel **G** shows the ORs and 95% confidence intervals for the top 10 ratio combinations of the 32-cytokines (based on adjusted *q*-value) when comparing BV-negative to BV-positive women that had IL-1β/IP-10 levels below the cohort median (*n* = 171 biologically independent samples). Source data are provided as a Source Data file.

Previous studies indicated that vaginal dysbiosis is associated with an innate immune response^32,44^. To further investigate the host immune landscape and bacterial vaginosis, we utilized the *molBV* tool to recapitulate categories of vaginal dysbiosis where other measures of BV were unavailable. We utilized 431 baseline samples from individual women participating in the placebo arm of the Costa Rica Vaccine Trial (CVT) that had 32 cytokine proteins (i.e., cytokines, chemokines and soluble receptors) quantitated from cervical secretions collected with a sponge (see Methods)^45^. Using three ordinal categories of BV derived from the *molBV* scores equivalent to Nugent BV negative (*molBV* 0–3, *n* = 179), intermediate (*molBV* 4–7, *n* = 70) and positive (*molBV* 7–10, *n* = 182), we identified 13 cytokines significantly associated with a trend across the three BV states (Fig. 2A, all markers *q* < 0.001). Cytokine levels were also tested with respect to age, smoking and HPV16 status and did not show any significant associations (Supplementary Table 2). In order to validate the use of ordinal BV categories, we performed additional sensitivity analyses using categorical BV states and found that the categorical models did not provide a better fit (Supplementary Fig. 4). The strongest positive association of ordinal *molBV* states was with IL-1β (unit increase OR = 1.73, 95% CI: 1.56–1.92), whereas IP-10 was inversely associated with BV (OR = 0.76, 95% CI: 0.68–0.85). Supplementary Table 3 shows the cytokines associated with *molBV*-BV in univariate analysis; 6 have been previously associated with BV and 7 additional cytokines are described in this report. Given that inflammation is a complex host response, we analyzed the interrelationships amongst the 32 cytokines to identify patterns of expression. A correlation network was constructed connecting those cytokines with a Pearson correlation >0.6 (see Fig. 2B). Since 18 of the markers show a strong correlation to at least one other marker, we sought to further improve the identification of differentially abundant inflammatory signals by using cytokine ratios that overcome some issues with compositional data and relative abundances^46^. Figure 2C shows a volcano plot indicating cytokine ratios (in red) with a *q* < 3.77*10^−44^ threshold. The ratios with the strongest affects were IL-1β/IP-10 and IP-10/TNF-α, shown at the far right and left, respectively. To further examine the relationships of cytokine ratios, we created a matrix showing the pairwise correlations associated with *molBV*-BV (Fig. 2D). Six highly correlated ratios share the IL-1β cytokine and 2 other highly correlated ratios share TNF-α (the 1st and 2nd highest ORs for a given cytokine, respectively, in the univariate analysis shown in Fig. 2A). Of the BV associated ratios, IL-1β/IP-10 had the strongest overall effect based on absolute odds ratio. Interestingly, IL-1β and IP-10 have been previously shown to be strongly associated with BV^47^, making this ratio very attractive for further consideration.

Exploring the distribution of the IL-1β/IP-10 ratio across the *molBV*-BV ordinal states revealed that despite the clear and consistent association (linear trend *p*-value = 3 × 10^−48^) with BV (Fig. 2E), 24% (85/349) of women with *molBV*-BV did not have elevated IL-1β/IP-10 levels. Whereas, surprisingly 28% (88/309) of BV negative women, had elevated IL-1β/IP-10 levels (see left violin plot, Fig. 2E). We used ANCOM to explore potential causes of *molBV*-negative women having high IL-1β/IP-10 levels; this analysis identified elevated levels of *L. iners* and *G. vaginalis*. Whereas, those with elevated levels of *L. crispatus* had low levels of IL-1β/IP-10 (W-stat threshold>10, FDR < 0.05, Fig. 2F).

To determine whether women with *molBV*-BV who did not show an elevated IL-1β/IP-10 signature had an alternative form of BV-associated inflammation, we compared this group to BV-negative women with similarly low levels of IL-1β/IP-10 (i.e., below the cohort median, Fig. 2G). The analysis revealed that TNF-α/MIP-1β was significantly positively associated with *molBV*-BV (OR = 1.24, 95% CI: 1.19–1.29) in the absence of elevated IL-1β/IP-10.

### Molecular BV, cervical inflammation, and the natural history of HPV

**Fig. 3:**
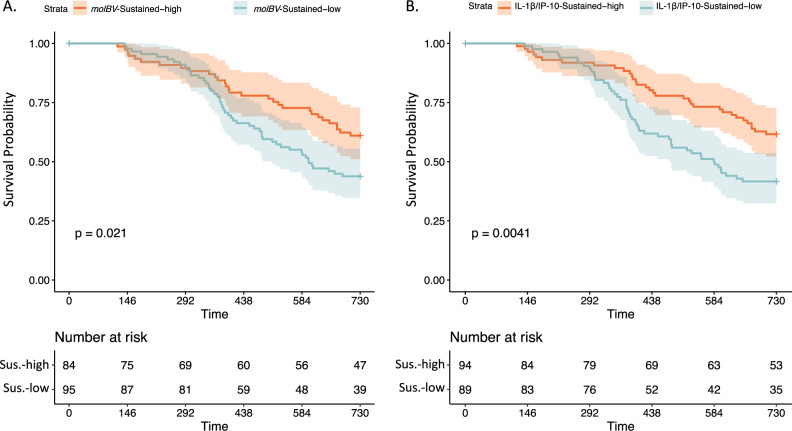
Molecular BV, inflammation, and HR-HPV clearance. Panel **A** shows the Kaplan–Meier curves for HR-HPV clearance colored by sustained BV status (i.e., having a *molBV* value above (red) or below (blue) the cohort median for both measured visits) with the unadjusted *p*-value presented in the bottom left corner of the plot and number of women at risk at each time point presented in the accompanying table directly below the plot. Panel **B** shows the Kaplan–Meier curves comparing women with either sustained high (red) or low (blue) IL-1β/IP-10 inflammation markers across the two analyzed visits. Sustained high and low IL-1β/IP-10 refers to women that had IL-1β/IP-10 levels above or below the cohort median for both measured visits, respectively, with the unadjusted *p*-value presented in the bottom left corner of the plot and number of women at risk at each time point presented in the accompanying table directly below the plot. Time is shown in days at the bottom of the figures and tables. Source data are provided as a Source Data file.

We previously reported that increased diversity of the cervicovaginal microbiome contributed to HPV natural history^16^. To evaluate the impact of bacterial vaginosis and directly test whether associated inflammation could be mediating the effect of BV on the natural history of HPV, we utilized the previously reported^16^ prospective cohort sub-study from the CVT trial. We utilized 16S NGS data from cervicovaginal DNA^16^ to calculate the *molBV* scores across two study visits (307/431 baseline participants had sequenced 16SV4 and cytokine data). Women who had sustained low levels of *molBV* vs. those that had sustained high *molBV* scores were more likely to clear HR-HPV over time (Fig. 3A, *p* = 0.02). Briefly, sustained levels refer to women that had *molBV* levels above or below the cohort median for both of the measured visits (203/307 were included). Similarly, sustained high-levels of BV-associated inflammation vs. low, as determined by IL-1β/IP-10, were associated with lower rates of HR-HPV clearance (Fig. 3B, *p* = 004). Sustained levels of this measure were also defined using stratification by the cohort median and agreement of the measure (above or below) at the two measured visits (183/307 were included). For detailed definitions of sustained-levels of *molBV* and IL-1β/IP-10 inflammation see the subsection “HPV natural history exposure/outcome definitions” in the “Methods” section of the manuscript.

To determine whether *molBV*-BV and/or sustained BV-associated inflammation markers acted as independent risk factors for HR-HPV clearance, we used cox-proportional hazard models after covariate adjustment. Table 1 presents the effects of *molBV* and IL-1β/IP-10 levels adjusted for age, smoking status and HPV16. Model 1 considered the dichotomized *molBV* states and found that compared to having sustained low levels of *molBV* (reference), the transition from high (V1) to low (V2) was a significant protective factor against clearing a HR-HPV infection (HR = 0.55, 95% CI: 0.31–0.97). Model 2 considered the effect of both IL-1β/IP-10 and *molBV* states. In this cytokine-adjusted analysis, *molBV* levels were not associated with HR-HPV clearance, suggesting that IL-1β/IP-10 was an independent driver of HPV clearance (HR = 1.87, 95% CI: 1.08–3.20). In this context, *molBV* states were not significant with the exception of the marginal signal from the group that became low at visit 2 (HR = 0.38, 95% CI: 0.15–1.00). Given the strong correlation between *molBV* and IL-1β/IP-10 levels (Fig. 2E), we made an additional parsimonious model (Model 3) to more accurately measure the effect of sustained low IL-1β/IP-10 levels on increasing the likelihood of HR-HPV clearance (HR = 1.86, 95% CI: 1.19–2.90). This model did not change the hazard ratio of sustained low IL-1β/IP-10 with HR-HPV clearance, but it did significantly reduce the *p*-value supporting the analyses that it is the true driver of HR-HPV clearance.

**Table 1:** Risk factors for time to clearance of incident HR-HPV infections.

	Model 1: *molBV*			Model 2: Sustained IL-1β/IP-10 and *molBV*			Model 3: Parsimonious		
Variable	HR	95% CI	*p* value	HR	95% CI	*p* value	HR	95% CI	*p* value
IL-1β/IP-10: Sustained-high ref)	—	—	—	—	—	—	—	—	—
IL-1β/IP-10: Sustained-low	—	—	—	**1.87**	**1.08–3.20**	**0.027**	**1.86**	**1.19–2.90**	**0.006**
Age	1.07	1.00–1.14	0.063	1.05	0.97–1.10	0.21	1.05	0.98–1.10	0.16
Smoking	0.87	0.66–1.14	0.32	0.90	0.64–1.30	0.53	0.90	0.64–1.30	0.53
HPV16 Status	1.04	0.73–1.49	0.82	0.97	0.61–1.60	0.91	0.98	0.61–1.60	0.91
*molBV*: Sustained-low (ref)	—	—	—	—	—	—	—	—	—
*molBV*: Became-high	0.84	0.51–1.38	0.49	0.97	0.52–1.80	0.94	—	—	—
*molBV*: Became-low	**0.55**	**0.31–0.97**	**0.039**	0.38	0.15–1.00	0.051	—	—	—
*molBV*: Sustained-high	0.85	0.56–1.28	0.43	1.05	0.57–1.90	0.88	—	—	—

This table shows the adjusted cox-proportional hazard models for the outcome of clearance of incident HR-HPV infections. In Model 1, categorical *molBV* state combinations from Visit 1 and Visit 2 are presented with adjustment for age, smoking status and HPV16 (at V2). In Model 2, sustained IL-1β/IP-10 levels are modeled with the inclusion of the molecular BV categories. For these analyses, both IL-1β/IP-10 and *molBV* were dichotomized as either high (above the median) or low (below the median) at each visit. Model 3 shows the adjusted hazard ratio of IL-1B/IP-10 levels without *molBV* in the model. Bolded values indicate statistically significant results (*p*-value < 0.05). HR, hazard ratio; 95% CI, 95% confidence interval. HR > 1.0 represent factors associated with a shorter time to clearance; whereas, HR < 1.0 are associated with increased time to clearance.

We next evaluated the association of BV and HR-HPV progression to CIN2+^16^. Briefly, the original study considered the binary outcome of persistent HR-HPV progressing to CIN2+ (diagnosed ~2 years after the second visit sample^16^) vs. HR-HPV infection clearance with CVM components serving as predictors. Using values from V2, we first tested whether molecular BV was associated with progression to CIN2+ using a generalized linear model (Table 2, Model 1). The results indicated that a continuous *molBV* score was prospectively associated with increased risk for CIN2+ progression with the odds of progression increasing by 1.24 (95% CI: 1.02–1.55) per unit increase of *molBV*. However, the dominant BV IL-1β/IP-10 signature was not significant when *molBV* was also in the model (Table 2, Model 2, OR = 1.15, 95% CI: 0.92–1.46). Remarkably, the alternative BV-associated inflammation signature represented by TNF-α/MIP-1β levels was a predictor of progression when either *molBV* (Table 2, Model 3, OR = 2.71, 95% CI: 1.46–5.61) or *molBV* and IL-1β/IP-10 were in the model (Table 2, Model 4, OR = 2.65, 95% CI: 1.39–5.68). Given the association of TNF-α/MIP-1β with *molBV* (Fig. 2C) and the modest correlation with IL-1β/IP-10 (Fig. 2D), an additional parsimonious model was constructed to more accurately measure the effect of TNF-α/MIP-1β with progression to CIN2+ without *molBV* or IL-1B/IP-10 (Table 2, Model 5, OR = 2.81, 95% CI: 1.65–5.42).

**Table 2:** Risk factors for progression to CIN2+.

Variable	Model 1: *molBV*			Model 2: IL-1β/IP-10 & *molBV*			Model 3: TNF-α/MIP-1β & *molBV*			Model 4: IL-1β/IP-10 and TNF-α/MIP-1β & *molBV*			Model 5: Parsimonious		
	OR	95% CI	*P* value	OR	95% CI	*P* value	OR	95% CI	*P* value	OR	95% CI	*P* value	OR	95% CI	*P* value
IL-1β/IP-10	—	—	—	1.15	0.92–1.46	0.22	—	—	—	1.04	0.80–1.34	0.73	—	—	—
TNF-α/MIP-1β	—	—	—	—	—	—	**2.71**	**1.46–5.61**	**0.002**	**2.65**	**1.39–5.58**	**0.004**	**2.81**	**1.62–5.42**	**0.0007**
*molBV*	**1.24**	**1.02–1.55**	**0.039**	1.16	0.92–1.48	0.23	1.03	0.81–1.31	0.82	1.01	0.78–1.32	0.92	—	—	—
Age	0.92	0.92–0.77	0.33	0.92	0.77–1.10	0.40	0.86	0.71–1.03	0.10	0.86	0.71–1.04	0.12	0.86	0.71–1.02	0.08
Smoking	1.47	0.66–3.23	0.33	1.51	0.67–3.36	0.31	1.37	0.59–3.14	0.54	1.39	0.59–3.21	0.53	1.35	0.59–3.05	0.56
HPV16 Status	1.26	0.48–3.29	0.63	1.27	0.48–3.35	0.67	1.62	0.58–4.69	0.44	1.60	0.57–4.65	0.45	1.63	0.58–4.70	0.44

This table shows the generalized linear models for the outcome of progression to CIN2+. Two inflammatory signatures were evaluated. Levels of IL-1β/IP-10 and TNF-α/MIP-1β were dichotomized into high or low based on above or below the median, respectively. Progression to CIN2+ was observed after the collected V2 sample for all subjects (average of 2.68 years after V2 sample collection). Variables measured at V2 were used for the analysis. Bolded values indicate statistically significant results (*p*-value < 0.05).

## Discussion

In this study, we develop a relatively simple means to characterize cervicovaginal samples for BV using 16S rRNA gene amplicon next-generation sequencing (NGS) data. It is well known that features of the CVM are strongly associated with BV^39,40,48–52^, and our study takes this relationship further and provides a quantitative score of 0–10, equivalent to the Nugent score. This method is particularly useful for high-throughput analyses to determine a “molecular” Nugent-like score (*molBV*-BV) in women that might not have been evaluated for BV, but have an available cervicovaginal sample. Development of this method used a stringent diagnosis of BV including subjects concurrent for BV by both Nugent and Amsel criteria. The method was validated in three additional cohorts suggesting the generalizability of this particular molecular approach to generate a Nugent-like score. Although it is known that there is substantial variation between the cervicovaginal microbiome between African women and women with European ancestry^53^, use of the *molBV* algorithm in two African populations revealed high diagnostic AUCs for BV of 0.97 and 0.88 for Soweto and Cape Town sets, respectively. Moreover, the *molBV* diagnostic was also used to evaluate the local host inflammatory response in a population without available BV measures (i.e., Nugent or Amsel). We identified a total of 13 different cytokines associated with *molBV*-BV, of which 7, had not been previously reported (Supplementary Table 3 and ref. ^32^). In addition, we utilize the *molBV* score and cytokine data to demonstrate the contribution of each to the natural history of HR-HPV infections using a prospective study design. Another interesting feature of this study was to demonstrate a prospective association of TNF-α with CIN2+ (previously reported in a cross-sectional study by Łaniewski et al. ^54^).

Cytokine ratios were used in order to better address the interrelated data structure and we observed that increasing IL-1β/IP-10 ratios were strongly correlated with increasing molecular BV scores (linear trend *p*-value = 3.67 × 10^−48^). IL-1β/IP-10 ratio was previously postulated to be a relevant signature for BV and the identification of women at higher risk of STI transmission^47^.

Despite a strong correlation between IL-1β/IP-10 and *molBV* states, there were still 24–28% of women that had an elevated ratio despite a *molBV score of 0–3*. Upon further analyses, elevated *L. iners* and *G. vaginalis* were identified in this enigmatic group. This finding is of interest since dominance of *Lactobacillus* species in the CVM is typically associated with vaginal health^40,49^. In the CVT cohort, *L. crispatus* was inversely associated with IL-1β/IP-10 inflammation consistent with previous reports^40^. It is of interest that women in the discovery set within a *Lactobacillus-dominated* clade and having clinical features of BV also had elevated levels of *L. iners* (Fig. 1A). It is possible that the association of *L. iners* with a BV-associated inflammatory state is due to strain-level variation since the biology of *L. iners* is perplexing^55–57^. This particular species of *Lactobacillus* differs from other members of the genus in many respects including genome characteristics that give it a more perplexing character as compared to other lactobacilli^56^. Furthermore, certain strains of *L. iners* appear to carry unique genes, such as those that encode for inerolysin^58^. France et al.^51^, postulated that these genes appear to have been horizontally transferred to *L. iners* from *G. vaginalis* and this allows certain members of this species to directly extract nutrients from host cells, which may explain how it can persist in a sub-optimal CVM and possibly induce vaginal inflammation. Deeper sequencing of the CVM will be required to validate this hypothesis. Alternatively, the community context of *L. iners* might influence its behavior and association with inflammation. It appears that specific bacteria could have context specific functionality depending on the observed molecular BV state at the time of sampling. Although the *molBV* algorithm was set to clinical parameters of BV, the current work suggests that additional stratification to identify women at risk for sub-clinical inflammation is necessary. This is especially important given the presented data showing the significance of these inflammatory shifts in the context of HR-HPV infections and possible implications for other diseases in which BV acts as a risk factor. Lastly, L. *iners* may reflect associations with other unmeasured determinants leading to BV^56^.

An alternative consideration that may play a role in the pathogenesis of BV, in addition to bacterial biomarkers is the importance of microbial biomass. Amplicon sequencing of the microbiome is compositional in nature and does not provide a direct means to establish exact microbial biomass^46^. This variable was previously shown to be relevant with the qPCR technique that allowed intermediate BV states to be resolved by quantifying bacterial loads of *G. vaginalis* and *A. vaginae*^59^. It would be interest to incorporate a quantitative technique such as the one developed by Morton et al. ^46^, to further expand molecular characterization of BV in future studies.

Another remarkable aspect of the cytokine analysis of this study is the heterogeneity of immune markers that were elevated across the strata of molecular BV states. It would be reasonable to predict that having an elevation of the predominant cytokine ration (i.e., IL-1β/IP-10) would yield similar distributions of other cytokines given the correlation levels observed in Fig. 2B and D. However, when comparing the molecular BV-positive to BV-negative women with low levels of IL-1β/IP-10, we observed that the BV-positive women had elevated levels of TNF-α/MIP-1β inflammatory cytokines. In fact, TNF-α/MIP-1β was the dominant signature elevated in molecular BV-positive women with low levels of IL-1β/IP-10. These results indicate that BV seems to be heterogeneous as to the exact type of sub-clinical host inflammatory response. Given the identifiable variance of inflammatory levels within individual strata of BV due to specific organisms such as *L. iners*, it is likely that specific taxa, or bacterial networks, might be associated with this observation. Further studies with deeper sequencing to identify the possible microbial genetic basis for these observations are needed.

There is increased interest in uncovering the relationship between the cervicovaginal microbiome and HPV natural history, since a number of studies show a correlation between vaginal microbial diversity, BV, and HPV clearance^16–20^. It is not currently known why certain high-risk infections clear while a small minority persists for years and eventually progresses to pre-cancer^60^. We utilized HR-HPV detection data within the Costa Rica vaccine trial cohort^45^ to evaluate cervical cytokine profiles, 16 S rRNA gene amplicon NGS data and the newly developed molecular BV states to interrogate possible mechanisms of HR-HPV infection clearance. Kaplan-Meier analyses revealed similar clearance patterns amongst women with sustained low vs. high IL-1β/IP-10 levels and low vs. high *molBV* scores (see Fig. 3), although the cytokine measure showed a stronger association with HR-HPV clearance. To better understand these relationships, we utilized Cox-proportional hazard modeling with adjustment for age, smoking status, and HPV16 infection. The association with *molBV*-BV was eliminated once we adjusted for an elevated IL-1β/IP-10 ratio, possibly indicating that a specific type of inflammation associated with BV was driving the relationship between HR-HPV persistence or clearance.

Another relevant HPV outcome is a progression of a persistent HR-HPV infection to pre-cancer (CIN2+)^58^. The CVM was previously reported to be predictive of this outcome^16,20,36,61^. However, when we tested the continuous IL-1β/IP-10 levels in a model, this cytokine signature did not add any additional information beyond *molBV* for HR-HPV progression. Surprisingly, when we tested the TNF-α/MIP-1β signature that is also associated with certain characteristics of *molBV*, we found that it was associated with CIN2+ progression even after adjustment for *molBV* as well as *molBV* and IL-1β/IP-10 (Table 2). The final analysis revealed that a single unit increase in the TNF-α/MIP-1β ratio was positively associated with an odds of 2.81 (95% CI: 1.65–5.42) of developing CIN2+ within 2-years of V2 sample collection. The data presented in the current report suggest that BV and the host response is a highly heterogeneous relationship and although BV is consistently associated with certain microbial shifts and overall community structure (e.g., higher alpha diversity), the host response can also be modified by the presence of specific taxa. These results may explain why certain studies do not see an association with CVM diversity (a surrogate for BV), but do see signals when analyzing specific bacteria^62^. Based on the observations reported in this study, these variations appear to have a substantial effect on the immune response, which in turn has an effect on HR-HPV clearance and progression to CIN2+.

The currently reported analyses have several weaknesses that should be taken into account when interpreting the data. All of the analyses of the CVM utilized 16S rRNA gene amplicon sequencing. This method limits the taxonomic resolution of bacteria and other organisms constituting the microbiome. A deeper exploration of BV using techniques such as shotgun metagenomics may provide a more thorough explanation as to why there is significant heterogeneity in the local host inflammatory response to BV amongst different women. Additionally, although our core analysis utilized compositionally aware approaches, rarefaction was used when calculating beta diversity, which may bias the magnitude of the PERMANOVA result. The relationships between *molBV* and the analyzed cytokines were considered in a linear context, other non-ordinal relationships might exist and are worthy of future investigation. Moreover, the developed *molBV* score was dependent on a relatively small set of bacteria and was based on “clean” BV diagnoses in which Amsel and Nugent’s tests agreed. This choice was made in order to facilitate the robustness of the measure. This appears to have been effective based on the associations seen within African populations but may present a limitation in populations with different structures of the CVM, especially ones that may occur in women where the two clinical scoring systems are discordant. Most of the women in the reported study are adolescents or young adults and we do not know if the analyses extend to women of all ages and geographic locations. Moreover, it is possible that other inflammatory signatures might exist in the cervicovaginal region that were not measured in the current report. Finally, in using the clinical Nugent score to guide our analyses we may have inadvertently missed important physiological phenomena of BV that are inherently diluted by this clinical score; future studies should utilize additional CVM reduction techniques such as the recently developed VALENCIA^51^, which produces community state types that are clinically agnostic.

Here we present a comprehensive molecular characterization of BV using 16S rRNA gene amplicon sequencing and a curated panel of cytokines. We demonstrate using multiple cohorts that 16S amplicon sequencing can be reliably used to diagnose BV employing the newly developed *molBV* score. We further demonstrate that this score was strongly correlated with a heterogeneous inflammatory landscape within the cervicovaginal region. Exploring these inflammatory markers further revealed a complex system of interactions between individual taxa, specific cytokines, and molecular BV states. In addition, we demonstrated that there is the potential for clinical relevance of the findings through the use of HR-HPV outcomes. We specifically show that different possible inflammatory states in BV are either associated with persistence of HR-HPV infection (i.e., IL-1β/IP-10) or the progression of infection to precancer (i.e., TNF-α/MIP-1β). In support of a role for TNF-α in the progression of HR-HPV infections, a recent report indicated that TNF-α was the main discriminatory biomarker associated with invasive cervical cancer^63^.

Whether the adverse health outcomes from BV are all based on the host inflammatory response remains to be rigorously evaluated. Deeper exploration of these associations is warranted using more robust techniques such as machine learning in order to further understand why certain women experience inflammation with BV, while others do not and how the host-microbiome relationships impact health. Lastly, the implication of BV inducing a local inflammatory response might imply signaling systemic inflammation, which was not assessed in this study. The role BV-induced inflammation might have in immune conditions more prevalent in women remains an interesting hypothesis that could have profound diagnostic and therapeutic ramifications.

## Methods

### Bacterial vaginosis training set

This component of the study was conducted within an ongoing HPV study at Mount Sinai Adolescent Health Center (MSAHC) in New York City^64,65^. Cervicovaginal samples were collected from female patients, 15–25 years of age, with vaginal symptoms suggestive of BV (*n* = 30) or no symptoms (*n* = 30), both groups were recruited sequentially from the same clinic. Pregnant women were excluded. The parent study and BV sub-study were approved by the Institutional Review Board at The Icahn School of Medicine at Mount Sinai.

### Diagnosis of bacterial vaginosis

Subjects were evaluated for Amsel criteria by the examining physician and the presence of 3 out of 4 Amsel criteria established a diagnosis of BV^66^. Vaginal swabs were collected for Nugent scores by carefully inserting a sterile swab into the vagina about two inches, gently rotating against the vaginal wall for 10–30 s, and then withdrawn without touching the skin to avoid contamination. De-identified swabs were placed in a plastic culturette tube and shipped overnight to an outside clinical laboratory for Nugent scoring following standardized criteria^24^. A composite score from 0 to 10 was generated with a diagnosis of Nugent BV assigned as follows: 0–3 was considered negative, 4–6 was considered intermediate, and 7–10 was considered diagnostic of BV.

### Microbiome sample collection and DNA extraction

Samples for microbiome analyses were collected using a Cytobrush^®^ placed in PreservCyt transport medium (ThinPrep^®^; Hologic, Marlborough, MA). Samples were stored immediately at −20 °C until transport to the research lab at the Albert Einstein College of Medicine. In the lab, the samples were transferred to a 15 ml tube and gently centrifuged at 1500 RPM for 5 min. After removing the supernatant by decanting, the pellets were rinsed in 3 ml of TE (10 mM Tris, 1.0 mM EDTA). This solution was then vortexed and centrifuged at 1500 RPM for 5 min and the supernatant was removed by decanting. The remaining pellet and leftover solution (~150 µl) were used for DNA isolation via column processing with the QIAamp Mini spin column (Qiagen, Valencia, CA) following the manufacturer’s protocol. The purified DNA was eluted in 150 µl of elution buffer (10 mM Tris/0.5 mM EDTA, pH 9).

### PCR amplification

PCR for bacterial communities was performed using forward (515F) GTGYCAGCMGCCGCGGTA and reverse (806R) GGACTACHVGGGTWTCTAAT primers which amplify the V4 hypervariable region of the prokaryotic 16S rRNA gene^67,68^. All primers contained unique Golay barcodes to allow for dual indexing of each sample. PCRs were conducted in a 25 µl reaction with 2 µl input of template DNA, 16.75 µl of ddH20, 2.5 µl of Platinum 10× PCR buffer (Invitrogen, Waltham, MA), 0.75 µl of MgCl_2_ (50 mM, Invitrogen), 0.5 µl of dNTP mix (10 mM each, Roche, Basel, Switzerland), 0.25 µl AmpliTaq Gold, polymerase (5 U/µl, Applied Biosystems, Carlsbad, CA), 0.25 µl of Platinum Taq DNA Polymerase (10 U/µl, Invitrogen), and 1 µl (5 µM) of each primer (IDT, Coralville, IA). Thermocycling conditions included an initial denaturation at 95 °C for 5 min, followed by 15 cycles of 95 °C for 1 m, 55 °C for 1 m, 72 °C for 1 m, followed by 15 cycles of 95 °C for 1 m, 60 °C for 1 m, 72 °C for 1 m, and a final extension at 72 °C for 10 min.

PCR for fungal communities was performed using barcoded forward (48F) ACACACCGCCCGTCGCTACT and reverse (217R) TTTCGCTGCGTTCTTCATCG primers that amplify the ITS1 region of the prokaryotic ribosomal gene cluster^38,69^. PCRs were conducted in a 25 µl reaction with 10 µl input of template DNA, 8.75 µl of ddH20, 2.5 µl of Platinum 10x PCR buffer (Invitrogen), 0.75 µl of MgCl_2_ (50 mM, Invitrogen), 0.5 µl of dNTP mix (10 mM each, Roche), 0.25 µl AmpliTaq Gold polymerase (5 U/µl, Applied Biosystems), 0.25 µl of Platinum Taq DNA Polymerase (10 U/µl, Invitrogen), and 1 µl (5 µM) of each primer (IDT, Coralville, IA). Thermocycling conditions included an initial denaturation at 95 °C for 5 mins, followed by 35 cycles of 95 °C for 30 s, 55 °C for 30 s, 72 °C for 2 min, followed by a final extension at 72 °C for 10 min. All PCRs were conducted in a GeneAmp PCR System 9700 (Applied Biosystems) and PCR products were verified by gel electrophoresis.

### Next Generation Library preparation and sequencing

PCR products for each sample were pooled by PCR assay (16S and ITS1) in approximately equal concentrations and 100 µl of the pooled products were loaded into a 3% agarose gel and run at 80 V for 3 h to separate the DNA fragments. The DNA fragment for each assay was excised and purified with a QIAquick Gel Extraction Kit (Qiagen) and quantified using a Qubit High Sensitivity dsDNA assay (Invitrogen). NGS library preparation was conducted on the purified pooled PCR samples from each assay with a KAPA LTP Library Preparation Kit (KAPA Biosystems, Wilmington, MA) according to the manufacturer’s protocol. The library amplicons were validated on a 2100 Bioanalyzer (Agilent Technologies, Santa Clara CA) and sequencing of libraries was carried out on an Illumina MiSeq with 2 × 300 bp paired-end reads kit at the Genomics Core of the Albert Einstein College of Medicine.

### Bioinformatics

Illumina reads were initially right trimmed to remove bases that fell below PHREAD score 25 using PRINSEQ-lite^70^. Reads were then demultiplexed using NovoBarcode based on unique dual Golay barcode combinations^71^.

QIIME2^72^ was used to identify amplicon sequence variants using DADA2^73^ for both the 16SV4 and ITS1 amplicon data. For 16SV4 amplicon sequence variants (ASVs) the naïve Bayesian classifier^74^ was used to assign taxonomy using the lab’s custom database that is comprised of GreenGenes 13.8^75^, HOMD^76^, and vaginal reference sequences^77^. For fungal taxonomic assignments, BLAST^78^ was used with the UNITE database^79^. Taxonomic assignment was combined with the ASV data using custom bash scripts into a biome file^80^ and further processed with R^81^.

### Statistical analysis

The *phyloseq*^82^ package was used to import microbiome data into R^83^ and to calculate the Chao1, Fisher and Shannon alpha diversity measures as well as the Jensen–Shannon diversity index for beta diversity analyses. The vegan^84^ package was used to run the PERMANOVA. The pROC package was used for the AUC analyses^85^. All data visualization was achieved using the ggplot2 package^86^.

The significance of belonging to taxon-specific hierarchical clusters in the heatmap analysis was assessed using Fisher’s exact test. Pairwise statistical significance in alpha diversity was determined using the Wilcoxon test. Significance in beta diversity was determined using PERMANOVA. ANCOM^87^ was used for bacterial taxa (i.e., biomarker) discovery. A linear model was used to determine significance of trends in the cytokine analyses and to extract ordinal ORs. Pearson coefficient was used for correlation analysis. The *q*-value^88^ package was used to correct the calculated linear trend *p*-values for multiple testing. Standard error of the mean was used to represent variation in sequencing depth between samples. Cox proportional hazard models were used in order to adjust the data for age, smoking, and HPV16 status in the survival analyses. The goodness of fit was assessed using the *gof* function from the *survMisc* package^89^ in all models and shown to have satisfactory performance at the 0.05 alpha threshold level. Age was treated as a continuous variable, HPV16 as binary (0 or 1) based on PCR results (V2), and smoking status as ordinal (0 = never smoker, 1 = former smoker, and 2 = current smoker) all were taken from V2.

### Calculating *molBV*

ANCOM^87^ was used to determine which bacterial genera were associated with BV through the use of microbial reference frames^46^ (Fig. 1E). Only those samples that were positive, or negative, for BV by both Amsel and Nugent criteria were used in the analysis (*n* = 18 BV-positive, *n* = 22 BV-negative). Out of the identified biomarkers only those that were present in ≥ 80% of samples with at least a 0.01% relative abundance after subsampling were retained for the calculation of *molBV*. These taxa included—*Lactobacillus, Prevotella, Gardnerella*, *Megasphaera*, *Parvimonas*, *Clostridium*, *Porphyromonas*, *Adlercreutzia*, *Dialister*, *Atopobium*, and *Sneathia*.

To create the microbial reference frames, log ratios were created using *Lactobacillus* and the markers elevated in the BV positive group (with *Lactobacillus* serving as the denominator in all ratios). The log ratios were then analyzed using a robust regression with the Nugent scores serving as the outcome and each ratio as the predictor. The beta coefficients and intercepts for each of the ratios were extracted and are presented in Supplementary Table 6.

To calculate *molBV*, which is the imputed continuous Nugent score, the log ratios between *Lactobacillus* and BV specific markers were generated (e.g., *Lactobacillus:Prevotella, Lactobacillus:Gardnerella, Lactobacillus: Shuttleworthia*, etc.) and used, along with the data from Supplementary Table 6, to calculate *molBV*:

$${molBV}=\frac{\mathop{\sum }\limits_{i}^{n}{{\beta }}_{{{{{\rm{0i}}}}}}+{{{\beta }_{1i}}X_{i}}}{n}$$

In the formula *X*_*i*_ represents the log ratio *i*, *β*_0*i*_ is the ratio’s corresponding intercept and *β*_1*i*_ is the beta coefficient. For an estimate to be valid for a given reference taxa, there had to be both *Lactobacillus* and a BV marker detected with a minimum of 1 read each. The final *molBV* score is the average of the valid log-ratio estimates that approximate the clinical Nugent score. Given the nature of regression prediction, *molBV* is not bound by the 0–10 range of the Nugent score and may take on non-integer values. To make the two scales more comparable, *molBV* was fit into the 0–10 range by using the following formula:

1 $${molBV}{\mbox{\_}}{scaled}=\frac{{molBV}-{{\min }}({molBV})}{{{\max }}\left({molBV}\right)-{{\min }}({molBV})}$$

In the formula *molBV* represents the raw score obtained from the above calculation, the max(*molBV*) is the highest calculated value in the cohort, the min(*molBV*) is the lowest calculated value in the cohort and *molBV_scaled* represents the final *molBV* score that falls into the desired 0–10 range.

Similar to Nugent scoring, ranges of the continuous *molBV* score, are used to define BV status. A *molBV* score of 0–3 is considered negative for BV, 4–6 is considered intermediate, and a score of 7–10 is considered consistent with BV.

### Confirmation cohorts

Three cohorts were used to confirm the *molBV* classifier. Full details about sample collection and processing can be obtained from the cited studies. The United States (USA) confirmation cohort was composed of 388 women with collection from three separate locations (two in Baltimore and one in Atlanta) with the women having a median age of 31 years^40^. The Cape Town cohort was composed of 90 women with a median age of 18 years^42,44^. The Soweto cohort was composed of 78 women with a median age of 18 years^42,44^.

### Cervical immune cytokines, chemokines, and soluble receptors

Cervical sponge samples were collected from women participating in the HPV Costa Rica Vaccine Trial (CVT) using a Merocel sponge (Medtronic Xomed, Jacksonville, FL) as previously described^90^. A customized panel including 32 cytokines, chemokines, and soluble receptors was quantitated using Luminex-based Milliplex Map Mulitplex Assays (Millipore, Billerica, MA) as previously described^91^.

### HPV natural history exposure/outcome definitions

DNA from cervical samples from the placebo arm of the Costa Rica vaccine trial^45^ were used to test the prospective association of molecular BV and cervicovaginal inflammation with HPV natural history stages. One analyzed outcome was time to clearance of high-risk HPVs (i.e., HPV16, 18, 31, 33, 35, 39, 45, 51, 52, 56, 58 and/or 59^92^). Women were selected from the CVT placebo arm where an incident high-risk HPV infection was detected (visit 1 sample this study). Clearance was recorded if a woman cleared all high-risk types detected at the incident visit within a 2-year observation window. The average time between collected-sample visits was 1.28 years. Data from the CVT trial^45^ was used to determine persistence status to ensure that the collected V2 sample fell within the observation window. The two core exposures were sustained high/low *molBV* and sustained high/low cervical inflammation as determined from IL-1β/IP-10 cytokine marker ratios. Inflammation and *molBV* sustained/persistent status were determined by median stratification; specifically the sustained status categories refer to a per-protocol approach where the exposure status had to be similar across the two analyzed visits. For example, the median *molBV* value across all study visits was 5.4 and if a women had a *molBV* value of 10 for visit 1 and 8 for visit 2, she would be placed in the sustained *molBV* high category. Participants with discordant *molBV* values were not included in this analysis in order to measure the per-protocol effect of sustained high/low *molBV* values in the context of HPV natural history (i.e., clearance or persistence). Excluded samples did not differ significantly from those retained in the analysis in terms of age, HPV16 positivity or smoking status (see Supplementary Tables 7.1 and 7.2). A second outcome analyzed was progression to CIN2+. CVT trial follow-up data was used to identify which of the study participants went on to develop CIN2+ after the V2 sample (average time to diagnosis was 2.68 years).

### Reporting summary

Further information on research design is available in the Nature Research Reporting Summary linked to this article.

## Supplementary information

Supplementary Information

Reporting Summary

## Data Availability

Sequence files and metadata for all samples used in this study have been uploaded to SRA (https://www.ncbi.nlm.nih.gov/bioproject/PRJNA641099). Script used to calculate *molBV* with instructions and sample test data can be found in GitHub (https://github.com/musyk07/molBV). Source data are provided with this paper.

## Source data

Source Data
