# Supplementary material for: molBV reveals immune landscape of bacterial vaginosis and predicts human papillomavirus infection natural history
Source: Nat Commun. 2022 Jan 11;13:233. doi: 10.1038/s41467-021-27628-3 (PMC8752746; doi:10.1038/s41467-021-27628-3)
Supplement: Supplementary file 1 — Supplementary Information [file 41467_2021_27628_MOESM1_ESM.pdf]

Supplementary Information

molBV Reveals Immune Landscape Of Bacterial Vaginosis and Predicts Human Papillomavirus Infection Natural History

Mykhaylo Usyk, Nicolas F. Schlecht, Sarah Pickering, LaShanda Williams, Christopher C. Sollecito, Ana Gradissimo, Carolina Porras, Mahboobeh Safaeian, Ligia Pinto, Rolando Herrero, Howard D. Strickler, Shankar Viswanathan, Anne Nucci-Sack, Angela Diaz, Costa Rica HPV Vaccine Trial (CVT) Group, Robert D. Burk

Contents

Supplementary Table 1. BV diagnosis agreement between Amsel and Nugent scores

Data is shown for 49 women where a definitive BV diagnosis using Nugent Score could be made. Not included are 8 cases diagnosed as intermediate by Nugent of whom 3 were positive and 5 were negative by Amsel's criteria.

Supplementary Table 2. Cytokine levels associated with age, smoking status and HPV16

The table shows the *P* values in the 2nd, 4th and 6th columns for age, smoking and HPV16 status using univariate linear regression with each cytokine serving as a continuous outcome, respectively. To account for multiple testing, *Q* values were calculated and presented in the table above in columns 3, 5 and 7.

Supplementary Table 3. Cytokines identified to be significantly associated with increasing *molBV*

Supplementary Table 4. IL-1β/IP-10 interaction with *molBV* (HR-HPV clearance outcome)

The table shows the interaction effects between categorical *molBV* states at V1 and V2 and IL-1β/IP-10 states as described in Table 1, Model 1. Outcome modeled is time to clearance of the incident HR-HPV infection identified at V1 as used in Table 1 (see Methods and Materials). None of the interaction terms were statistically significant. HR, hazard ratio; CI, 95% confidence interval.

Supplementary Table 5. Cytokine interactions with *molBV* (progression to CIN2+ Outcome)

Table shows the interaction effects between *molBV* and two cytokine ratios as shown in Table 2, Model 2 and Model 3. The first model considers the interaction with IL-1β/IP-10 and the 2nd with TNF-α/MIP-1β. The outcome measured is progression to CIN2+ status after the V2 sample collection (average of 2.68 years after V2 sample collection). Neither interaction terms reached statistical significance.

Supplementary Table 6. Beta coefficients and intercepts for microbial reference frames used to calculate *molBV*

Supplementary Table 7.1 Characteristics of individuals with *molBV* concordance vs. discordance between V1 and V2

Supplementary Table 7.2 Characteristics of individuals with IL-1β/IP-10 concordance vs. discordance between V1 and V2

Supplementary Fig. 1. Heatmap showing clustering of the cervicovaginal microbiome based on the top 20 fungal species

Heatmap shows the top 20 fungal species. The two rows directly below the dendrogram show the sample case status based on Nugent and Amsel criteria in the first and second row, respectively (labeled at left of heatmap). The color coding of these rows is shown in the key to the left of the heatmap. The fungal taxa are shown to the right of the heatmap.

Supplementary Fig. 2. Clinical BV vs. molecular fungal alpha and beta diversity

Panel A shows the alpha diversity differences of the Chao1, Fisher and Shannon diversity indices for BV diagnosed by Amsel or Nugent criteria. Significance is shown at the top of each panel and calculated using two-sided unpaired Wilcoxon ranked sum test. The middle of each box plot represents the median, the lower whisker represents the minimum of the data, the top whisker represents the maximum, the notches around the median represent the 95% confidence interval of the median, the box itself represents the interquartile range (50% of the data) and points on top or below the whiskers are the outliers. The diversity metric is shown to the right of each set of figures. Panel B shows beta diversity analysis using PCoA and Jensen-Shannon diversity index for the Amsel BV diagnosis (not significant). Panel C shows beta diversity analysis using PCoA and Jensen-Shannon diversity index for the Nugent BV diagnosis (not significant).

Supplementary Fig. 3. Benchmarking molecular diagnoses of BV vs. clinical BV diagnosis

A. The calculated molecular BV score (*molBV*) is shown on the y-axis vs. the clinical Nugent score (x-axis) for each of the three tested cohorts (USA, Cape Town and Soweto). Intraclass Correlation Coefficient (ICC) was used to assess the consistency between *molBV* and the Nugent scores and is shown at the top of the cohort panels. B., C. and D. show the Area Under the Curve (AUC) analyses for diagnosing BV using *molBV* and three measures of the CVM (Chao1/Shannon alpha diversity and Lactobacillus abundance) within the USA, Cape Town and Soweto cohorts, respectively. In panels B-D each line shows the AUC analysis for each component. Specific AUC values are provided above the figure panels.

Supplementary Figure 4. Model Checking Using Categorical *molBV* States

The Forest plot shows the ORs and associated 95% Confidence Intervals of cytokines found to be significantly associated with ordinal *molBV* states modeled as categorical exposures with BV-negative serving as the reference group and being compared to BV-intermediate and BV-positive groups. To the right of the plot are the anova analyses that compared the new categorical model to the corresponding ordinal regression from Figure 2C. An insignificant p-value indicates that the categorical data does not fit the data better than the ordinal groups.

Supplementary Information

		Amsel's Criteria	
		BV(+)	BV(-)
Nugent Score	BV (+)	18	8
	BV (-)	1	22

Supplementary Table 1. BV diagnosis agreement between Amsel and Nugent scores

Data is shown for 49 women where a definitive BV diagnosis using Nugent Score could be made. Not included are 8 cases diagnosed as intermediate by Nugent of whom 3 were positive and 5 were negative by Amsel's criteria.

Supplementary Table 2. Cytokine levels associated with age, smoking status and HPV16

Cytokine	age_pval	age_Qval	smoking_pval	smoking_Qval	hpv16_pval	hpv16_Qval
SGP130	0.019	0.307	0.423	1.000	0.241	0.997
GRO	0.019	0.307	0.234	1.000	0.502	0.997
SCD40L	0.072	0.697	0.937	1.000	0.670	0.997
IFN_G	0.110	0.697	0.786	1.000	0.557	0.997
IL_6	0.120	0.697	0.141	1.000	0.969	0.997
SIL_4R	0.144	0.697	0.881	1.000	0.832	0.997
IL_8	0.170	0.697	0.800	1.000	0.491	0.997
G_CSF	0.176	0.697	0.833	1.000	0.221	0.997
TGFA	0.196	0.697	0.940	1.000	0.416	0.997
STNFRI	0.218	0.699	0.930	1.000	0.950	0.997
IL_1A	0.293	0.852	0.525	1.000	0.789	0.997
TNF_A	0.320	0.852	0.475	1.000	0.838	0.997
MIP_1B	0.477	0.970	0.251	1.000	0.148	0.997
VEGF	0.481	0.970	0.681	1.000	0.424	0.997
IL_7	0.529	0.970	0.192	1.000	0.877	0.997
STNFRII	0.532	0.970	0.517	1.000	0.964	0.997
IFNA_2	0.550	0.970	0.605	1.000	0.997	0.997
SIL_6R	0.558	0.970	1.000	1.000	0.910	0.997
IP_10	0.642	0.970	0.996	1.000	0.078	0.997
SVEGFR1	0.716	0.970	0.829	1.000	0.260	0.997
SVEGFR2	0.718	0.970	0.589	1.000	0.164	0.997
IL_1RA	0.725	0.970	0.969	1.000	0.656	0.997
MCP_1	0.731	0.970	0.878	1.000	0.459	0.997
IL_1B	0.732	0.970	0.882	1.000	0.746	0.997
IL_15	0.785	0.970	0.541	1.000	0.873	0.997
SVEGFR3	0.863	0.970	0.928	1.000	0.845	0.997
EOTAXIN	0.867	0.970	0.508	1.000	0.430	0.997
IL_10	0.897	0.970	0.393	1.000	0.583	0.997
EGF	0.924	0.970	0.419	1.000	0.109	0.997
SEGFR	0.959	0.970	0.928	1.000	0.697	0.997
IL_2	0.960	0.970	0.866	1.000	0.992	0.997
SILRII	0.970	0.970	0.769	1.000	0.826	0.997

The table shows the *P* values in the 2nd, 4th and 6th columns for age, smoking and HPV16 status using univariate linear regression with each cytokine serving as a continuous outcome, respectively. To account for multiple testing, *Q* values were calculated and presented in the table above in columns 3, 5 and 7.

Supplementary Table 3. Cytokines identified to be significantly associated with increasing *molBV*

Cytokine	Odds Ratio	Categorical trend p-val	Categorical trend q-val	Citing Literature	Cytokine Description
**IL-1B**	1.73 [1.56-1.92]	2.98E-22	9.54E-21	Positive<br>Nonpregnant women [1-9]<br>Pregnant [10-14]<br>Pregnant black women [15]<br>Undergoing IVF treatment [16]	Inflammation/cell proliferation, differentiation, apoptosis
**IL-1A**	1.52 [1.36-1.69]	3.43E-13	9.95E-12	Positive<br>Nonpregnant women [8]<br>Pregnant [17]<br>Twin pregnancies [18]	Acute phase protein/infection response
**SEGFR**	1.64 [1.48-1.81]	2.85E-19	8.83E-18		Cell migration/ adhesion/proliferation/mutations of receptor in cancer
**STNFRI**	1.5 [1.34-1.68]	2.93E-12	7.61E-11		Inflammatory marker of chronic disease
**SIL-6R**	1.51 [1.35-1.68]	8.69E-13	2.35E-11		Trans-signaling of IL-6 via gp130
**IL-15**	1.36 [1.22-1.52]	7.33E-08	1.83E-06		Regulates T cell and NK cell activation and proliferation
**TNF-A**	1.58 [1.42-1.77]	2.67E-15	8.00E-14	Positive<br>Nonpregnant women [1,19]<br>Pregnant [10]	Acute phase protein/infection response
**SVEGFR1**	1.32 [1.18-1.48]	2.31E-06	5.31E-05		Inhibits lymphangiogenesis
**SILRII**	1.31 [1.17-1.46]	5.62E-06	1.24E-04		
**IP-10**	0.76 [0.68-0.85]	1.83E-06	4.38E-05	Negative<br>Nonpregnant women [8]<br>Pregnant white women [15]<br>No Association<br>Nonpregnant women [20]	Anti-tumor chemoattractant
**IL-10**	1.55 [1.38-1.74]	4.63E-13	1.30E-11	Positive<br>Nonpregnant women [21]	Immunosuppressive & anti-inflammatory cytokine
**SCD40L**	1.27 [1.14-1.43]	2.67E-05	5.61E-04		Induces pro-inflammatory tumoridical cytokines/B cell activation
**IL-2**	1.28 [1.14-1.43]	3.39E-05	6.77E-04	Positive<br>Nonpregnant women [21]	White blood cell regulator

*** Data for only the significantly associated immune markers is shown (after adjustment for multiple testing, q<0.05, 15/32 tested markers). The full list of analyzed markers included: EGF, EOTAXIN, GRO, G_CSF, IFNA_2, IFN_G, IL_10, IL_15, IL_1A, IL_1B, IL_1RA, IL_2, IL_6, IL_7, IL_8, IP_10, MCP_1, MIP_1B, SCD40L, SEGFR, SGP130, SILRII, SIL_4R, SIL_6R, STNFRI, STNFRII, SVEGFR1, SVEGFR2, SVEGFR3, TGFA, TNF_A and VEGF.**

Supplementary Table 4. IL-1β/IP-10 interaction with *molBV* (HR-HPV clearance outcome)

Variable	HR	95% CI	P val
IL-1β/IP-10: Sustained-high (ref)	-	-	-
IL-1β/IP-10: Sustained-low	1.2383	0.47-3.24	0.66
Age	1.0573	0.97-1.15	0.19
Smoking	0.7656	0.53-1.1	0.14
HPV16 Status	1.0381	0.65-1.65	0.87
molBV: Sustained-low (ref)	-	-	-
molBV: Became-high	0.4934	0.12-2.08	0.34
molBV: Became-low	0.1583	0.02-1.37	0.09
molBV: Sustained-high	0.7201	0.27-1.91	0.51
IL-1β/IP-10: Sustained-low * *molBV*: Became-high	2.3048	0.47-11.33	0.30
IL-1β/IP-10: Sustained-low * *molBV*: Became-low	4.2846	0.36-50.66	0.25
IL-1β/IP-10: Sustained-low * *molBV*: Sustained-high	1.1591	0.31-4.34	0.83

The table shows the interaction effects between categorical *molBV* states at V1 and V2 and IL-1β/IP-10 states as described in Table 1, Model 1. Outcome modeled is time to clearance of the incident HR-HPV infection identified at V1 as used in Table 1 (see Methods and Materials). None of the interaction terms were statistically significant. HR, hazard ratio; CI, 95% confidence interval.

Supplementary Table 5. Cytokine interactions with *molBV* (progression to CIN2+ Outcome)

	***molBV* * IL-1β/IP-10interaction**			**molBV * TNF-α/MIP-1β interaction**		
**Variable**	**OR**	**95% CI**	**P val**	**OR**	**95% CI**	**P val**
IL-1β/IP-10	1.22	0.63-2.49	0.56	-	-	-
TNF-a/MIP-1B	-	-	-	1.52	0.3-9.01	0.62
molBV	1.13	0.82-1.61	0.46	1.23	0.72-2.13	0.45
Age	0.92	0.77-1.1	0.36	0.86	0.7-1.03	0.11
Smoking	1.53	0.67-3.48	0.3	1.37	0.58-3.14	0.46
HPV16 Status	1.28	0.48-3.42	0.61	1.62	0.57-4.71	0.37
IL-1β/IP-10* *molBV*	0.99	0.89-1.09	0.85		-	-
TNF-α/MIP-1β * *molBV*	-	-	-	1.11	0.83-1.49	0.46

Table shows the interaction effects between *molBV* and two cytokine ratios as shown in Table 2, Model 2 and Model 3. The first model considers the interaction with IL-1β/IP-10 and the 2nd with TNF-α/MIP-1β. The outcome measured is progression to CIN2+ status after the V2 sample collection (average of 2.68 years after V2 sample collection). Neither interaction terms reached statistical significance.

Supplementary Table 6. Beta coefficients and intercepts for microbial reference frames used to calculate *molBV*

Reference Frame	beta	intercept
log(Lactobacillus/Prevotella)	-1.0305	7.2775
log(Lactobacillus/Gardnerella)	-1.0012	6.7448
log(Lactobacillus/Megasphaera)	-1.6761	24.9232
log(Lactobacillus/Parvimonas)	-0.8007	10.2133
log(Lactobacillus/Clostridium)	-1.2528	10.0125
log(Lactobacillus/Porphyromonas)	-0.8971	10.8979
log(Lactobacillus/Adlercreutzia)	-1.0922	9.9193
log(Lactobacillus/Dialister)	-1.1281	9.6347
log(Lactobacillus/Atopobium)	-1.2970	9.8496
log(Lactobacillus/Sneathia)	-0.9494	8.3180

Supplementary Table 7.1 Characteristics of individuals with *molBV* concordance vs. discordance between V1 and V2

Variables	*molBV* Concordant	*molBV* Discordant	OR (95% CI)	*P* value
N	327	104		
visit_age Mean ± SD	23.03 ± 2.65	22.66 ± 2.5	-	0.244
HPV16				
Negative	218 (66.7%)	67 (64.4%)	1.1 (0.67 - 1.79)	0.722
Positive	109 (33.3%)	37 (35.6%)	-	-
Smoking_cat				
Never	249 (76.1%)	73 (70.2%)	-	-
Former	48 (14.7%)	18 (17.3%)	0.78 (0.42 - 1.52)	0.428
Current	30 (9.2%)	13 (12.5%)	0.68 (0.32 - 1.49)	0.338

Supplementary Table 7.2 Characteristics of individuals with IL-1β/IP-10 concordance vs. discordance between V1 and V2

Variables	IL-1β/IP-10 Concordant	IL-1β/IP-10 Discordant	OR (95% CI)	*P* value
N	307	124		
visit_age Mean ± SD	22.9 ± 2.69	23.06 ± 2.41	-	0.42
HPV16				
Negative	203 (66.1%)	82 (66.1%)	1 (0.63 - 1.59	1
Positive	104 (33.9%)	42 (33.9%)	-	-
Smoking_cat				
Never	227 (73.9%)	95 (76.6%)	-	-
Former	48 (15.6%)	18 (14.5%)	1.12 (0.6 - 2.15)	0.768
Current	32 (10.4%)	11 (8.9%)	1.22 (0.57 - 2.79)	0.721

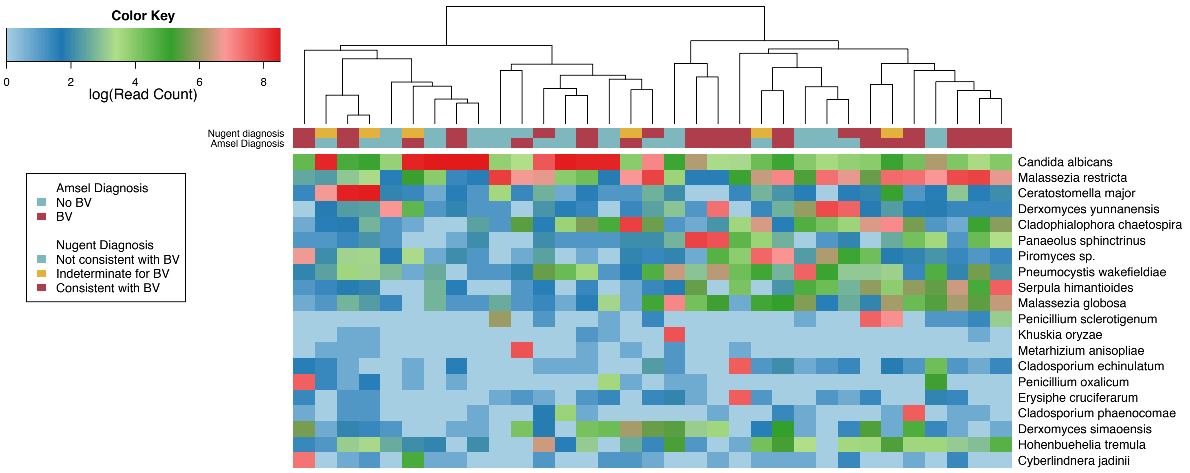

Supplementary Fig. 1. Heatmap showing clustering of the cervicovaginal microbiome based on the top 20 fungal species

Heatmap shows the top 20 fungal species. The two rows directly below the dendrogram show the sample case status based on Nugent and Amsel criteria in the first and second row, respectively (labeled at left of heatmap). The color coding of these rows is shown in the key to the left of the heatmap. The fungal taxa are shown to the right of the heatmap.

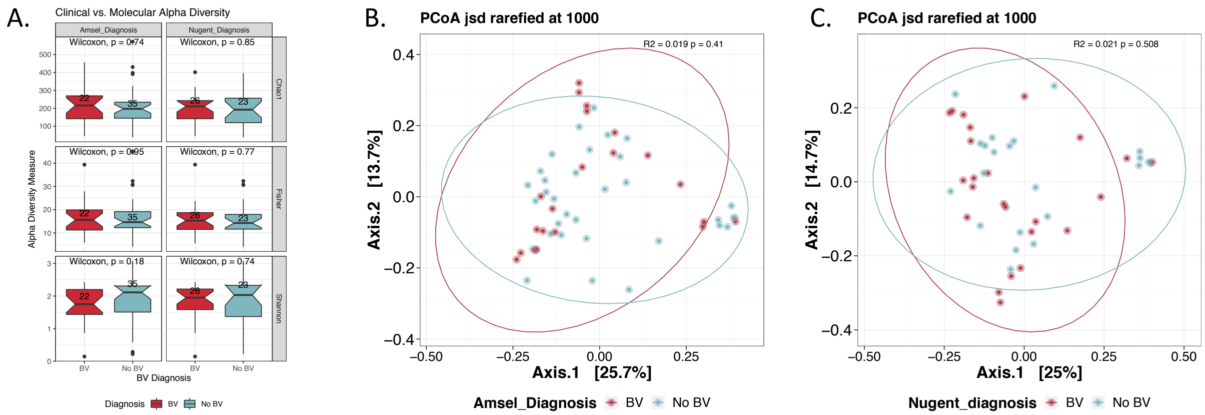

Supplementary Fig. 2. Clinical BV vs. molecular fungal alpha and beta diversity

Panel A shows the alpha diversity differences of the Chao1, Fisher and Shannon diversity indices for BV diagnosed by Amsel or Nugent criteria. Significance is shown at the top of each panel and calculated using two-sided unpaired Wilcoxon ranked sum test. The middle of each box plot represents the median, the lower whisker represents the minimum of the data, the top whisker represents the maximum, the notches around the median represent the 95% confidence interval of the median, the box itself represents the interquartile range (50% of the data) and points on top or below the whiskers are the outliers. The diversity metric is shown to the right of each set of figures. Panel B shows beta diversity analysis using PCoA and Jensen-Shannon diversity index for the Amsel BV diagnosis (not significant). Panel C shows beta diversity analysis using PCoA and Jensen-Shannon diversity index for the Nugent BV diagnosis (not significant).

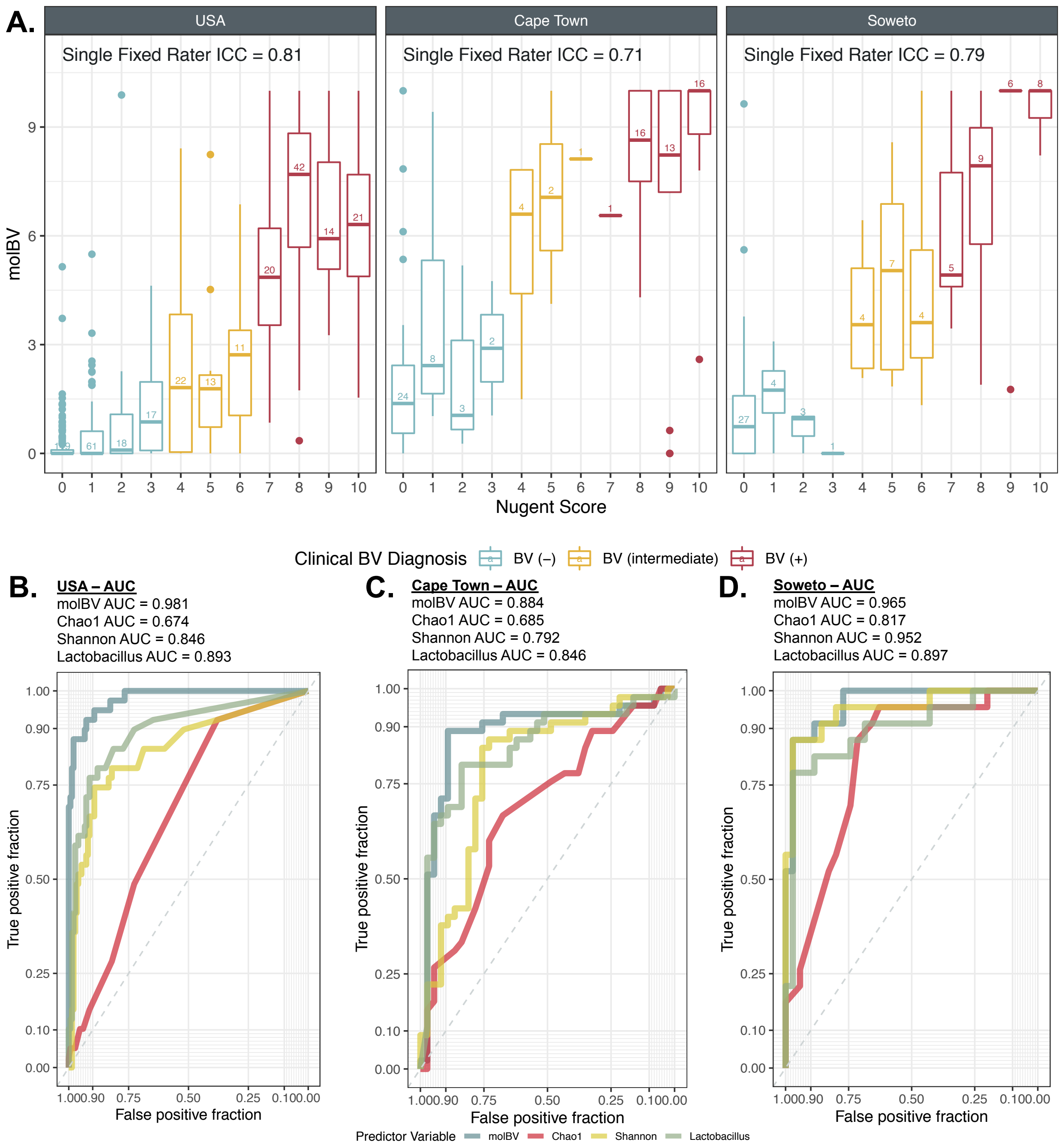

Supplementary Fig. 3. Benchmarking molecular diagnoses of BV vs. clinical BV diagnosis

A. The calculated molecular BV score (*molBV*) is shown on the y-axis vs. the clinical Nugent score (x-axis) for each of the three tested cohorts (USA, Cape Town and Soweto). Intraclass Correlation Coefficient (ICC) was used to assess the consistency between *molBV* and the Nugent scores and is shown at the top of the cohort panels. B., C. and D. show the Area Under the Curve (AUC) analyses for diagnosing BV using *molBV* and three measures of the CVM (Chao1/Shannon alpha diversity and Lactobacillus abundance) within the USA, Cape Town and

Soweto cohorts, respectively. In panels B-D each line shows the AUC analysis for each component. Specific AUC values are provided above the figure panels.

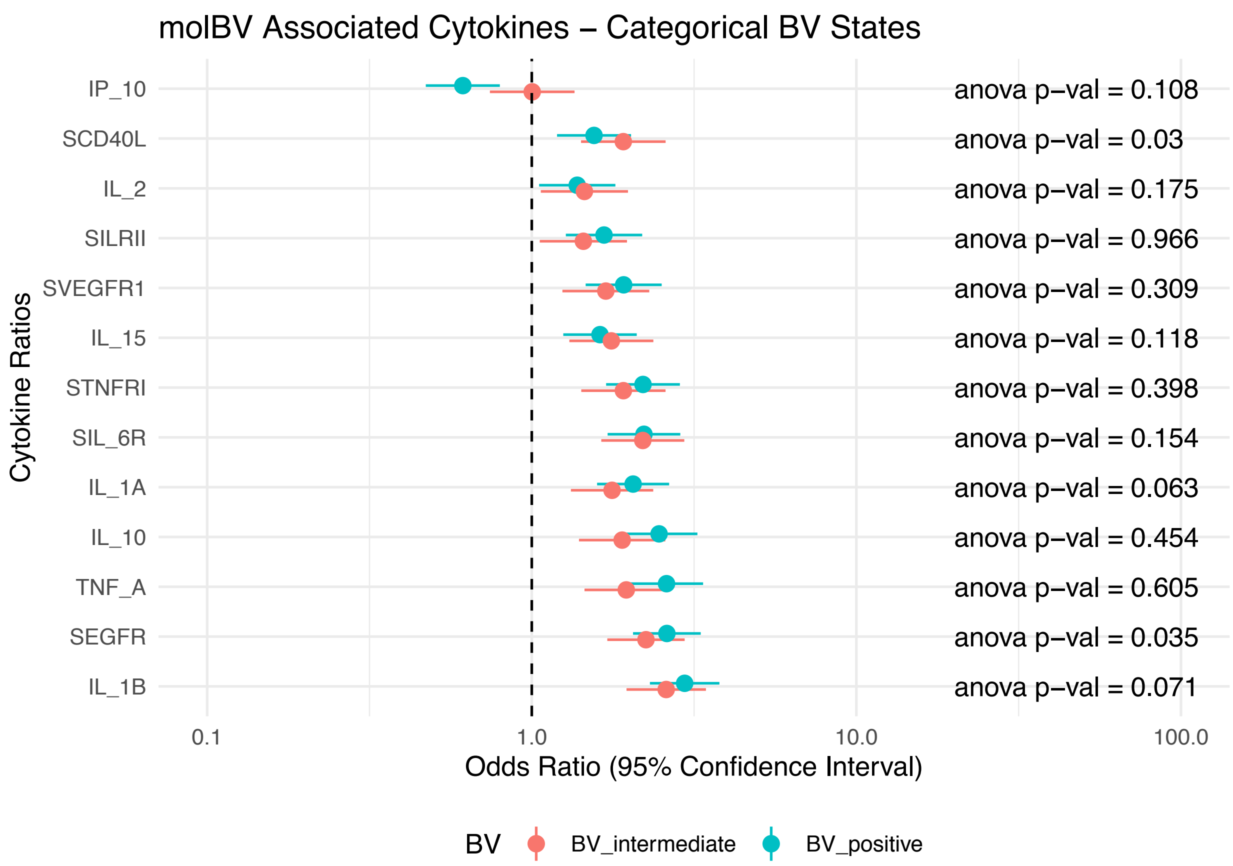

Supplementary Figure 4. Model Checking Using Categorical *molBV* States

The Forest plot shows the ORs and associated 95% Confidence Intervals of cytokines found to be significantly associated with ordinal *molBV* states modeled as categorical exposures with BV-negative serving as the reference group and being compared to BV-intermediate and BV-positive groups. To the right of the plot are the anova analyses that compared the new categorical model to the corresponding ordinal regression from Figure 2C. An insignificant p-value indicates that the categorical data does not fit the data better than the ordinal groups.

Supplementary Reference:

1 Sturm-Ramirez, K., Gaye-Diallo, A., Eisen, G., Mboup, S. & Kanki, P. J. High Levels of Tumor Necrosis Factor—α and Interleukin-1β in Bacterial Vaginosis May Increase Susceptibility to Human Immunodeficiency Virus. *The Journal of Infectious Diseases* **182**, 467-473, doi:10.1086/315713 (2000).

2 Cauci, S. *et al.* Interrelationships of interleukin-8 with interleukin-1β and neutrophils in vaginal fluid of healthy and bacterial vaginosis positive women. *Molecular Human Reproduction* **9**, 53-58, doi:10.1093/molehr/gag003 (2003).

3 Anton, G., Rid, J., Mylonas, I., Friese, K. & Weissenbacher, E. R. Evidence of a TH1-Shift of Local Vaginal Inflammatory Response During Bacterial Vaginosis. *Infection* **36**, 147-152, doi:10.1007/s15010-007-7152-2 (2008).

4 Cauci, S., Driussi, S., Guaschino, S., Isola, M. & Quadrifoglio, F. Correlation of Local Interleukin-1beta Levels with Specific IgA Response Against Gardnerella vaginalis Cytolysin in Women with Bacterial Vaginosis. *American Journal of Reproductive Immunology* **47**, 257-264, doi:10.1034/j.1600-0897.2002.01096.x (2002).

5 Hedge, S. R., Barrientes, F., Desmond, R. A. & Schwebke, J. R. Local and Systemic Cytokine Levels in Relation to Changes in Vaginal Flora. *The Journal of Infectious Diseases* **193**, 556-562, doi:10.1086/499824 (2006).

6 Spandorfer, S. D., Neuer, A., Giraldo, P. C., Rosenwaks, Z. & Witkin, S. S. Relationship of abnormal vaginal flora, proinflammatory cytokines and idiopathic infertility in women undergoing IVF. *J Reprod Med* **46**, 806-810 (2001).

7 Mitchell, C. M. *et al.* Bacterial Vaginosis, Not HIV, Is Primarily Responsible for Increased Vaginal Concentrations of Proinflammatory Cytokines. *AIDS Research and Human Retroviruses* **24**, 667-671, doi:10.1089/aid.2007.0268 (2008).

8 Masson, L. *et al.* Defining genital tract cytokine signatures of sexually transmitted infections and bacterial vaginosis in women at high risk of HIV infection: a cross-sectional study. *Sex Transm Infect* **90**, 580-587 (2014).

9 Hemalatha, R. *et al.* Cervicovaginal Inflammatory Cytokines and Sphingomyelinase in Women With and Without Bacterial Vaginosis. *The American Journal of the Medical Sciences* **344**, 35-39, doi:https://doi.org/10.1097/MAJ.0b013e318235597b (2012).

10 Mattsby-Baltzer, I., Platz-Christensen, J. J., Hosseini, N. & Rosen, P. IL-1beta, IL-6, TNFalpha, fetal fibronectin, and endotoxin in the lower genital tract of pregnant women with bacterial vaginosis. *Acta Obstet Gynecol Scand* **77**, 701-706 (1998).

11 Imseis, H. M. *et al.* Characterization of the inflammatory cytokines in the vagina during pregnancy and labor and with bacterial vaginosis. *J Soc Gynecol Investig* **4**, 90-94 (1997).

12 Cauci, S., Culhane, J. F., Di Santolo, M. & McCollum, K. Among pregnant women with bacterial vaginosis, the hydrolytic enzymes sialidase and prolidase are positively associated with interleukin-1β. *American Journal of Obstetrics and Gynecology* **198**, 132.e131-132.e137, doi:https://doi.org/10.1016/j.ajog.2007.05.035 (2008).

13 Beigi, R. H., Yudin, M. H., Cosentino, L., Meyn, L. A. & Hillier, S. L. Cytokines, Pregnancy, and Bacterial Vaginosis: Comparison of Levels of Cervical Cytokines in Pregnant and Nonpregnant Women with Bacterial Vaginosis. *The Journal of Infectious Diseases* **196**, 1355-1360, doi:10.1086/521628 (2007).

14 Basso, B., Giménez, F. & López, C. IL-1b, IL-6 and IL-8 levels in gyneco-obstetric infections. *Infectious diseases in obstetrics and gynecology* **13**, 207-211 (2005).

15 Ryckman, K. K., Williams, S. M., Krohn, M. A. & Simhan, H. N. Racial differences in cervical cytokine concentrations between pregnant women with and without bacterial vaginosis. *Journal of reproductive immunology* **78**, 166-171 (2008).

16 Boomsma, C. M. *et al.* Is bacterial vaginosis associated with a pro-inflammatory cytokine profile in endometrial secretions of women undergoing IVF? *Reproductive BioMedicine Online* **21**, 133-141, doi:https://doi.org/10.1016/j.rbmo.2010.03.022 (2010).

17 Platz-Christensen, J. J., Mattsby-Baltzer, I., Thomsen, P. & Wiqvist, N. Endotoxin and interleukin-1 alpha in the cervical mucus and vaginal fluid of pregnant women with bacterial vaginosis. *Am J Obstet Gynecol* **169**, 1161-1166, doi:10.1016/0002-9378(93)90274-m (1993).

18 Wennerholm, U. B. *et al.* Interleukin-1alpha, interleukin-6 and interleukin-8 in cervico/vaginal secretion for screening of preterm birth in twin gestation. *Acta Obstet Gynecol Scand* **77**, 508-514 (1998).

19 Zariffard, M. R. *et al.* Induction of Tumor Necrosis Factor–α Secretion and Toll-Like Receptor 2 and 4 mRNA Expression by Genital Mucosal Fluids from Women with Bacterial Vaginosis. *The Journal of Infectious Diseases* **191**, 1913-1921, doi:10.1086/429922 (2005).

20 Weissenbacher, T. *et al.* Interleukin-6, interleukin-10 and interleukin-12 in vaginal fluid from women with bacterial vaginosis. *Archives of gynecology and obstetrics* **281**, 77 (2010).
