# Supplementary material for: molBV reveals immune landscape of bacterial vaginosis and predicts human papillomavirus infection natural history
Source: Nat Commun. 2022 Jan 11;13:233. doi: 10.1038/s41467-021-27628-3 (PMC8752746; doi:10.1038/s41467-021-27628-3)
Supplement: Supplementary file 2 — Reporting Summary [file 41467_2021_27628_MOESM2_ESM.pdf]

nature portfolio

Corresponding author(s): Mykhaylo Usyk, Robert Burk

Last updated by author(s): Nov 10, 2021

Reporting Summary

Nature Portfolio wishes to improve the reproducibility of the work that we publish. This form provides structure for consistency and transparency in reporting. For further information on Nature Portfolio policies, see our Editorial Policies and the Editorial Policy Checklist.

Statistics

For all statistical analyses, confirm that the following items are present in the figure legend, table legend, main text, or Methods section.

n/a	Confirmed	
☐	☒	The exact sample size (n) for each experimental group/condition, given as a discrete number and unit of measurement
☐	☒	A statement on whether measurements were taken from distinct samples or whether the same sample was measured repeatedly
☐	☒	The statistical test(s) used AND whether they are one- or two-sided<br>*Only common tests should be described solely by name; describe more complex techniques in the Methods section.*
☐	☒	A description of all covariates tested
☐	☒	A description of any assumptions or corrections, such as tests of normality and adjustment for multiple comparisons
☐	☒	A full description of the statistical parameters including central tendency (e.g. means) or other basic estimates (e.g. regression coefficient) AND variation (e.g. standard deviation) or associated estimates of uncertainty (e.g. confidence intervals)
☐	☒	For null hypothesis testing, the test statistic (e.g. F, t, r) with confidence intervals, effect sizes, degrees of freedom and P value noted<br>*Give P values as exact values whenever suitable.*
☒	☐	For Bayesian analysis, information on the choice of priors and Markov chain Monte Carlo settings
☒	☐	For hierarchical and complex designs, identification of the appropriate level for tests and full reporting of outcomes
☐	☒	Estimates of effect sizes (e.g. Cohen's d, Pearson's r), indicating how they were calculated

Our web collection on statistics for biologists contains articles on many of the points above.

Software and code

Policy information about availability of computer code

Data collection	All data generated in the manuscript uses common software/tools that are cited within the manuscript. Data collection for disease outcomes were performed using clinicians and informed consent as indicated in the methods section of the manuscript.

Data analysis	Bioinformatics<br>Illumina reads were initially right trimmed to remove bases that fell below PHREAD score 25 using PRINSEQ-lite (version 0.20.4). Reads were then demultiplexed using NovoBarcode (version 1.00) based on unique dual Golay barcode combinations. QIIME version 2 was used to identify amplicon sequence variants using DADA2 (version 1.16) for both the 16SV4 rRNA and ITS1 amplicon data. For 16SV4 rRNA amplicon sequence variants (ASVs) the naïve Bayesian classifier (version 11.5) was used to assign taxonomy using the lab's custom database that is comprised of GreenGenes (version 13.8), HOMD (version 9.14) and vaginal reference sequences. For fungal taxonomic assignments, BLAST (version legacy 2.2.26) was used with the UNITE database (version 8.3). Taxonomic assignment was combined with the ASV data using phyloseq (version 1.28.0) and further processed with R (version 3.6.1).<br><br>Statistical Analysis<br>The phyloseq package (version 1.28.0) was used to import microbiome data into R (version 3.6.1) and to calculate the Chao1, Fisher and Shannon alpha diversity metrics as well as the Jensen-Shannon diversity index for beta diversity analysis. The vegan package (version 2.5-6) was used to run the PERMANOVA. The pROC package (version 1.16.12) was used for the AUC analysis. All data visualization was achieved using the ggplot2 package (version 3.3.2) .<br>Significance of belonging to taxon-specific hierarchical clusters in the heatmap analysis was assessed using Fisher's exact test. Pairwise statistical significance in alpha diversity was determined using the Wilcoxon test. Significance in beta diversity was determined using PERMANOVA. ANCOM (version 2.0) was used for bacterial taxa (i.e., biomarker) discovery. A linear model was used to determine significance of trends in the cytokine analyses and extract ordinal ORs. Pearson coefficient was used for correlation analysis. The q-value package (version 2.16.0) was used to correct the calculated linear trend p-values for multiple testing.

For manuscripts utilizing custom algorithms or software that are central to the research but not yet described in published literature, software must be made available to editors and reviewers. We strongly encourage code deposition in a community repository (e.g. GitHub). See the Nature Portfolio guidelines for submitting code & software for further information.

Data

Policy information about availability of data

All manuscripts must include a data availability statement. This statement should provide the following information, where applicable:

- Accession codes, unique identifiers, or web links for publicly available datasets
- A description of any restrictions on data availability
- For clinical datasets or third party data, please ensure that the statement adheres to our policy

Data Availability
Sequence files and metadata for all samples used in this study have been uploaded to SRA (https://www.ncbi.nlm.nih.gov/bioproject/PRJNA641099). Script used to calculate molBV with instructions and sample test data can be found in GitHub (https://github.com/musyk07/molBV). Source data are provided with this paper.

Field-specific reporting

Please select the one below that is the best fit for your research. If you are not sure, read the appropriate sections before making your selection.

☒ Life sciences ☐ Behavioural & social sciences ☐ Ecological, evolutionary & environmental sciences

For a reference copy of the document with all sections, see nature.com/documents/nr-reporting-summary-flat.pdf

Life sciences study design

All studies must disclose on these points even when the disclosure is negative.

Sample size	For the development of the molBV score 60 women chosen based on clinical BV status determined using Amsel's criteria to have 30 BV+ and 30 BV- cases. Sample size was chosen to be sufficiently large to capture the normal distribution within the datasets (i.e. 30 for cases and 30 for controls). All subsequent cohorts were used fully to maximize statistical power (i.e. we used all available samples within each cohort). For the USA cohort 388 subjects were used; for Cape Town 90 subjects were used; for Soweto 78 subjects were used; and for Costa Rica (prospective HPV outcome testing) 431 subjects were used with testing across 2 study visits.
Data exclusions	In the original Mt. Sinai datasets 3 women were excluded due to inadequate data collection. The subjects did not differ from the full set and were thus not expected to introduce differential loss bias.
Replication	Replication from the original dataset was performed across 3 large cohorts. All cohorts confirmed that 16S NGS data can be used to consistently identify clinical BV cases. AUCs for all cohorts with accompanying ROC curves provided in the manuscript. Briefly the USA cohort had an AUC of 0.98 and the Cape Town and Soweto Cohorts had AUCs of 0.88 and 0.97 respectively.
Randomization	Original cases were selected from the Mount Sinai Adolescent Health Center study upon presentation with BV. Controls were selected upon admission using incidence density sampling to represent the same risk set as cases. All confirmation cohorts used random population sampling from specific age groups pertinent to their original study designs. For full details please refer to the original study descriptions cited in the methods section of the main text.
Blinding	Cases were screened for BV status upon admission into the cohort, so blinding was not possible due to the nature of clinical presentation of BV.

Reporting for specific materials, systems and methods

We require information from authors about some types of materials, experimental systems and methods used in many studies. Here, indicate whether each material, system or method listed is relevant to your study. If you are not sure if a list item applies to your research, read the appropriate section before selecting a response.

Materials & experimental systems

n/a	Involved in the study
☒	☐ Antibodies
☒	☐ Eukaryotic cell lines
☒	☐ Palaeontology and archaeology
☒	☐ Animals and other organisms
☐	☒ Human research participants
☐	☒ Clinical data
☒	☐ Dual use research of concern

Methods

n/a	Involved in the study
☒	☐ ChIP-seq
☒	☐ Flow cytometry
☒	☐ MRI-based neuroimaging

Human research participants

Policy information about studies involving human research participants

Population characteristics	This study was conducted within an ongoing HPV study at Mount Sinai Adolescent Health Center (MSAHC) in New York City. Cervicovaginal samples were collected from female patients, 15 to 25 years of age, with vaginal symptoms suggestive of BV (n=30) or no symptoms (n=30), both groups were recruited sequentially from the same clinic. Pregnant women were excluded.
Recruitment	Participants were recruited during routine screening visits of an ongoing HPV vaccine trial. This aspect is a major strength of the study as it does not rely on participants having symptoms of BV, which would bias selection towards more severe cases of BV. We therefore do not expect self-selection bias due to severity of BV to play a factor in our results.
Ethics oversight	The parent study and BV sub-study were approved by the Institutional Review Board at The Icahn School of Medicine at Mount Sinai.

Note that full information on the approval of the study protocol must also be provided in the manuscript.

Clinical data

Policy information about clinical studies

All manuscripts should comply with the ICMJEguidelines for publication of clinical research and a completedCONSORT checklist must be included with all submissions.

Clinical trial registration	ClinicalTrials.gov NCT00128661
Study protocol	Full trial protocol is cited within the main text of the manuscript.
Data collection	This study was conducted within an ongoing HPV study at Mount Sinai Adolescent Health Center (MSAHC) in New York City, New York (state), USA. The samples were collected between clinic visits ranging from 11/8/2018 - 6/19/2017.
Outcomes	The primary outcome of clinical BV were determined using Amsel's composite criteria and the Nugent score by a trained physician.
